# Editorial: Relationships between plant disease and microbiomes

**DOI:** 10.3389/fpls.2023.1285682

**Published:** 2023-09-25

**Authors:** Jiangang Li

**Affiliations:** Institute of Soil Science, Chinese Academy of Sciences, Nanjing, China

**Keywords:** plant health, soil- borne diseases, soil microbe, soil health, plant nutrient

It is well known that a wide variety of microorganisms colonize plant tissues in both managed and natural ecosystems. Among these plant-associated microorganisms, soil-borne pathogens are a major threat to agricultural production. Common pathogenic genera include *Fusarium*, *Rhizoctonia*, *Ralstonia*, *Verticillium*, and *Phytophthora*. Soil-borne diseases such as seedling damping-off, root rot, and wilting have caused massive decline and devastated entire agricultural industries. Almost every country or geographical region in the world faces problems with soil-borne diseases, and some soil-borne diseases have been a serious problem for decades and pose limitations to agricultural yields. (Mehrdad and Matthew, 2023).

A large number of microorganisms live in the soil. In addition to plants, microorganisms living in soil and deep surface have the largest biomass on earth. The content of microbial biomass carbon usually exceeds 0.5 mg. Therefore, soil can be considered to be the main source of microorganisms in terrestrial ecosystems where bacteria are most abundant. Such diverse microbial communities have direct and indirect effects on the health and well-being of soil, plants, animals, and humans (Banerjee and van der Heijden, 2023).

Direct benefits conferred by microorganisms to host plants include protection from plant pathogens through competition for nutrients and niches, production of antibiotics and hydrolytic enzymes. The impact of the soil microbiome on maintaining plant health is most clearly demonstrated in disease-suppressive soils, where soil microorganisms inhibit disease occurrence by acting as the first line of defense against soil-borne pathogens through antagonism. Although the interactions between microorganisms and the specific pathogens responsible for disease suppression are biologically complex, the mechanisms in charge of building- up of disease- suppressive soils are similar, including production of antifungal metabolites by different bacterial genera and antagonism for beneficial microbes against pathogens.

Biological control of plant diseases is also a powerful example of soil microorganisms promoting plant health. A number of effective biological control techniques utilize live bacterial preparations. They are an important component of the microbiota of agroecosystems and play a positive role for plants in habitat adaptation and resistance to biotic and abiotic stresses. For example, more than 80 species of soil fungi can promote plant growth and suppress soil-borne diseases (Tariq et al., 2020).

In addition to direct effects on harmful rhizosphere microorganisms, some beneficial microorganisms have been shown to capable of inducing plant immune responses, such as induced systemic resistance (ISR) (Berendsen et al., 2012). Moreover, management practices such as crop rotation and the addition of organic fertilizers are also used to drive microbiome changes to achieve general or specific disease suppression.

Soil microbiomes have important functions in improving plant nutrition. Of the 29 essential plant elements, 18 come from the soil and are transported to plant tissues by soil microorganisms. The molecular mechanisms underlying the symbiotic relationship between plants and *arbuscular mycorrhizal* fungi (AMF) have been well documented. AMF can forms symbiotic relationships with nearly 90% of land plants that can rely on AMF or their symbiotic endophytes to meet their phosphorus needs under low inorganic phosphate conditions.

The importance of the soil microbiome to plant health is well understood, and there is growing evidence that plants can determine the composition of their microbiomes in the recent years. Some key results were published in this Research Topic. Li et al. compared the severity of plant diseases caused by *Fusarium proliferatum* invasion in different seasons of bamboo. They found that pathogen presence correlated with seasonal changes in the bamboo root microbiome and decreased bacterial richness in diseased plants. Sun et al. assessed the difference of bacterial and fungal community composition treated by azoxystrobin between tobacco healthy and diseased leaves. Liu et al. functionally characterized the MoMih1 in *Magnaporthe oryzae* and demonstrated that MoMih1 was an important regulator required for the fungal development and plant infection of *M. oryzae*. Fan et al. reported chitin amendments remarkably increased soil pH and available nutrients, improved soybean plant growth and soil microbial activities (FDA) in diseased soil induced by continuous cropping of soybean for five years. Moreover, chitin application significantly enriched the relative abundances of the potential biocontrol bacteria and fungi. Together, all of these articles emphasized the importance of soil microbiome to plant health.

More research is needed to deeply understand the complex interactions between plants and rhizosphere microorganisms. It is hoped that many new insights on microbiome processes as well as identify and isolate microorganisms important for soil and plant health will be brought in the near future, which will open new avenues to increase crop quality and yield.

## Author contributions

JL: Writing – original draft, Writing – review & editing.
